# Supplementary material for: MetaRibo-Seq measures translation in microbiomes
Source: Nat Commun. 2020 Jun 29;11:3268. doi: 10.1038/s41467-020-17081-z (PMC7324362; doi:10.1038/s41467-020-17081-z)
Supplement: Supplementary file 10 — Supplementary Data 7 [file 41467_2020_17081_MOESM10_ESM.zip › File2/Confidence_VeryHigh_Taxonomy/293489_out.krona.html]

Javascript must be enabled to view this page.

members
magnitude
magnitudeUnassigned
count
unassigned
taxon
rank

293489\_out

23

23
superkingdom
2

23
1239
phylum

23
class
186801

23
186802
order

186803
family
20

1
genus
841

1
301301

SRS146812\_contig\_number\_20632
species

19
189330

SRS147653\_contig\_number\_contig-100\_18764.18765
1
genus

2
1896999

SRS148253\_contig\_number\_contig-100\_858.859SRS148721\_contig\_number\_47483
species

species
1263074

SRS017521\_contig\_number\_49065SRS148159\_contig\_number\_41555SRS149879\_contig\_number\_27631
3

species

SRS015217\_contig\_number\_28371SRS015431\_contig\_number\_81571SRS015782\_contig\_number\_28080SRS019068\_contig\_number\_90515SRS020869\_contig\_number\_29183SRS049712\_contig\_number\_contig-100\_700.135279SRS053356\_contig\_number\_42513SRS064276\_contig\_number\_19885SRS077392\_contig\_number\_contig-100\_1646.222514SRS1041039\_contig\_number\_10117SRS104636\_contig\_number\_4383SRS143991\_contig\_number\_22209SRS144537\_contig\_number\_contig-100\_28719.78570
88431
13

541000
family
3

3
genus
1263

3
species
165186

SRS043768\_contig\_number\_contig-100\_3413.184850SRS103987\_contig\_number\_34239SRS148159\_contig\_number\_7738
